# Supplementary material for: Label-Free Quantitative Proteomics Reveal the Involvement of PRT6 in Arabidopsis thaliana Seed Responsiveness to Ethylene
Source: Int J Mol Sci. 2022 Aug 19;23(16):9352. doi: 10.3390/ijms23169352 (PMC9409418; doi:10.3390/ijms23169352)
Supplement: Supplementary file 1 [file ijms-23-09352-s001.zip › Supplementary Figures S1, S2 and S3 and Tables S1 and S2.pdf]

Supplementary results

Figure S1. Dot plot of enriched BP terms in Col+/Col− (A) and *prt*6−/Col− (B) determined from GSEA results.

Figure S2. GSEA plot showing enrichment of lipid localization category among protein expressions in *prt6*− versus Col−

Figure S3. Individual pathway deregulation scores (PDS) of the selected biological process related to hormone response.

Table S1. Differentially expressed proteins in comparison groups after Tukey analysis of 619 proteins in Col+, Col−, prt6+, prt6−.

Table S2. Proteins present in the 30 h-proteome belonging to the category of lipid localization.

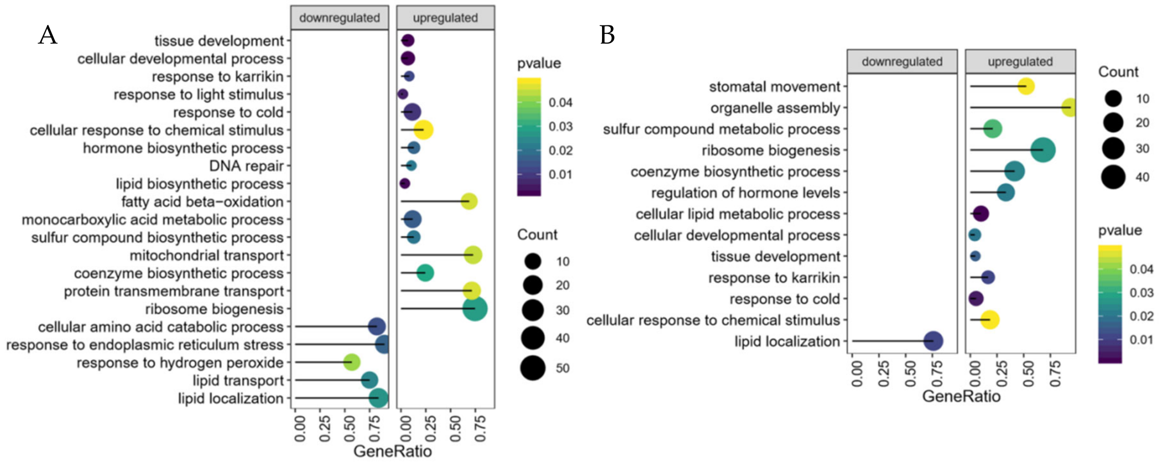

Figure S1. Dot plot of enriched BP terms in Col+/Col− (A) and *prt*6−/Col− (B) determined from GSEA results. The size of the dot represents count of the core proteins in each set, the color represents p-value, and generatio represents the number of core genes in gene set/the total number of genes in a gene set.

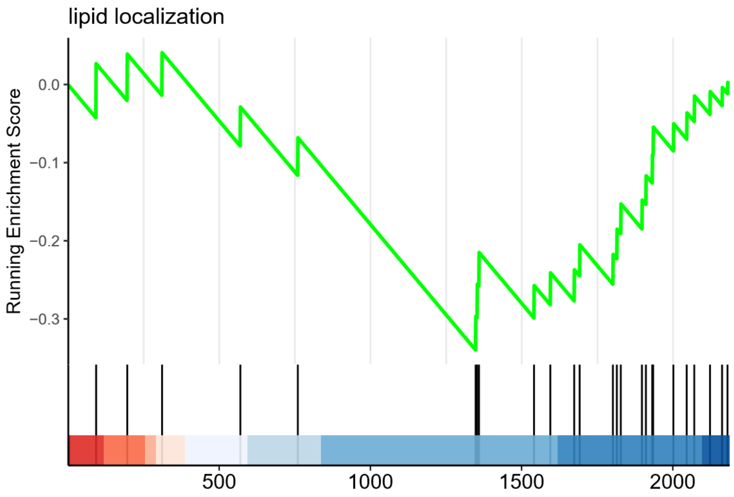

Figure S2. GSEA plot showing enrichment of lipid localization category among protein expressions in *prt6−* versus Col−. The green line represents the running enrichment score; the color bar from red to blue represents the descending order of the ratio (prt6−/Col−); the short black lines corresponds to the presence of members in this BP category; the leading gene set contains the proteins appear subsequent to the peak score.

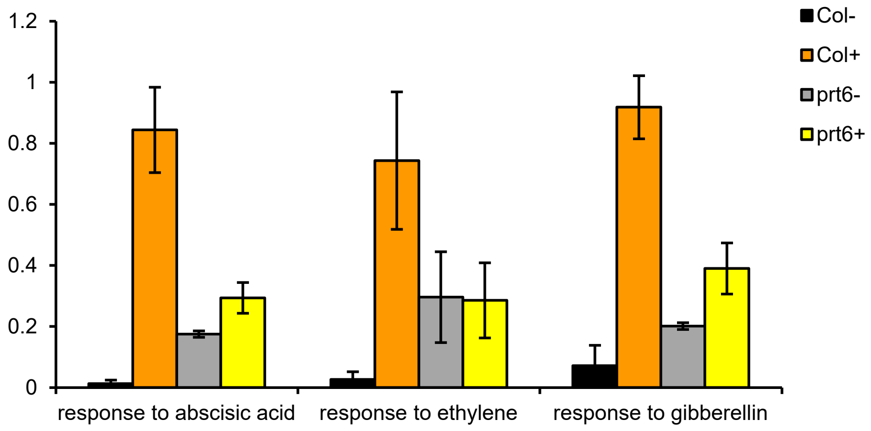

Figure S3. Individual pathway deregulation scores (PDS) of the selected biological process related to hormone response.

Table S1. Differentially expressed proteins in comparison groups after Tukey analysis of 619 proteins in Col+, Col−, prt6+, prt6−. Down and up-regulated proteins were determined by P value (< 0.05) and fold change (< 0.7 or > 1.3).

Tukey analysis	down	up	total
Col+/Col−	311	276	587
prt6+/prt6−	15	15	30
prt6−/Col−	23	77	100

Table S2. Proteins present in the 30 h-proteome belonging to the category of lipid localization.

ID	Category	Col+/Col−	prt6+/prt6−	prt6−/Col−
AT1G31812	acyl-CoA binding protein	0.76	0.74	1.09
AT5G53470	acyl-CoA binding protein	0.57	1.68	0.53
AT3G05420	acyl-CoA binding protein	1.16	1.12	1.13
AT5G59310	lipid-transfer protein	0.50	0.95	0.98
AT2G38530	lipid-transfer protein	0.49	0.75	0.83
AT1G27950	lipid-transfer protein	0.87	0.93	0.86
AT4G30880	lipid-transfer protein	0.64	0.76	0.90
AT5G38160	lipid-transfer protein	0.53	0.88	0.79
AT5G38170	lipid-transfer protein	0.63	1.04	1.24
AT5G55410	lipid-transfer protein	0.51	0.60	0.98
AT5G42890	sterol carrier protein	2.24	0.91	0.97
AT4G27140	Albumin	0.79	0.76	0.82
AT4G27150	Albumin	0.70	0.66	1.57
AT4G27160	Albumin	0.72	1.54	0.33
AT4G27170	Albumin	0.78	0.89	0.87
AT5G54740	Albumin	0.45	0.66	0.77
AT1G48990	Oleosin	0.55	0.76	0.91
AT2G25890	Oleosin	0.74	0.72	0.86
AT3G01570	Oleosin	0.51	1.01	0.66
AT3G18570	Oleosin	0.64	0.93	0.94
AT4G25140	Oleosin	0.86	1.40	0.93
AT5G40420	Oleosin	0.60	0.88	0.84
AT3G27660	Oleosin	0.58	1.03	0.74
AT1G05510	Oli body associated protein	0.58	0.95	0.82
AT1G01050	Pyrophosphorylase	2.02	1.31	1.35
